# First person – Mikiko Oka

**DOI:** 10.1242/dmm.052388

**Published:** 2025-04-29

## Abstract

First Person is a series of interviews with the first authors of a selection of papers published in Disease Models & Mechanisms, helping researchers promote themselves alongside their papers. Mikiko Oka is first author on ‘ Glucose uptake in pigment glia suppresses Tau-induced inflammation and photoreceptor degeneration’, published in DMM. Mikiko conducted the research described in this article while a graduate student in Kanae Ando's lab at the Tokyo Metropolitan University, Hachioji, Tokyo, Japan. She is now a postdoc in the lab of Shinya Yamamoto at Baylor College of Medicine, Houston, TX, USA, investigating age-associated human diseases by using *Drosophila* models.


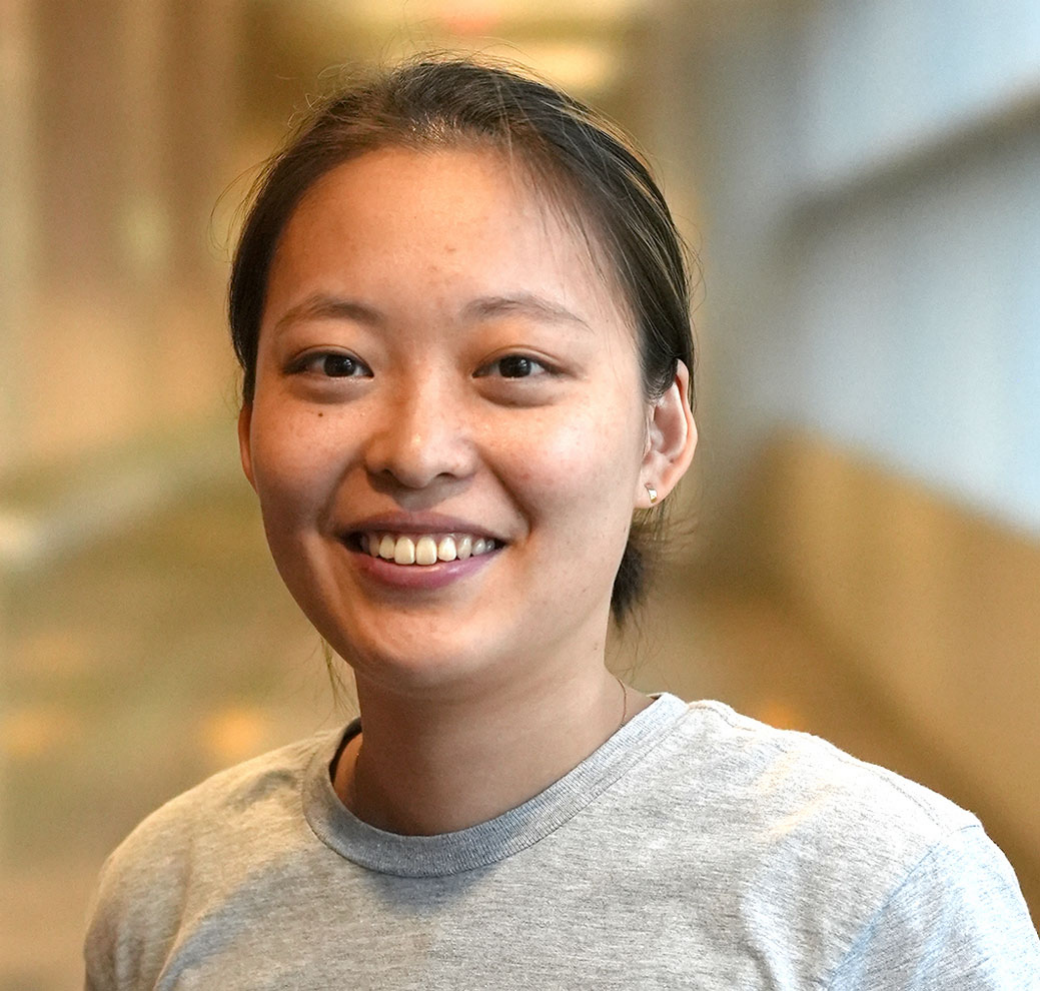


**Mikiko Oka**

**Who or what inspired you to become a scientist?**

Kanae Ando, my mentor during my PhD training. She showed me how much fun science can be and became my first female role model of a research scientist.

**What is the main question or challenge in disease biology you are addressing in this paper? How did you go about investigating your question or challenge?**

We aimed to uncover the role of glucose metabolism in the pathogenesis of Alzheimer's disease (AD), which is characterized by amyloid plaques of amyloid beta protein and neurofibrillary tangles of Tau protein. Although disruptions in glucose metabolism have been linked to AD, the specific contributions of glucose metabolism to the disease's progression remain poorly understood. By using our preferred model animal *Drosophila melanogaster*, we genetically enhanced glucose uptake in a tissue-specific manner to observe how glucose metabolism affects degeneration caused by Tau.

When glucose uptake was increased in glial cells […] it suppressed the hyperinflammatory status and ameliorated degeneration caused by Tau.

**How would you explain the main findings of your paper to non-scientific family and friends?**

Glucose metabolism is crucial for brain functions, but disruptions in this process have been observed in the brain of AD patients. In these brains, hyperinflammation and Tau protein aggregation are associated with neuronal cell death. In our study, we investigated how glucose metabolism is involved in AD pathogenesis caused by Tau. By using a fruit fly model expressing human Tau − a well-established AD model − we increased glucose uptake by expressing glucose transporters in a cell-type-specific manner. When glucose uptake was increased in glial cells, which are essential for supporting neurons and maintaining brain homeostasis, it suppressed the hyperinflammatory status and ameliorated degeneration caused by Tau.

**What are the potential implications of these results for disease biology and the possible impact on patients?**

Our study provides new insights into how proper glucose metabolism in glial cells plays a vital role in the immune system and helps prevent neurodegenerative diseases.

I always prefer to publish my results in Open Access journals.

**Figure:**
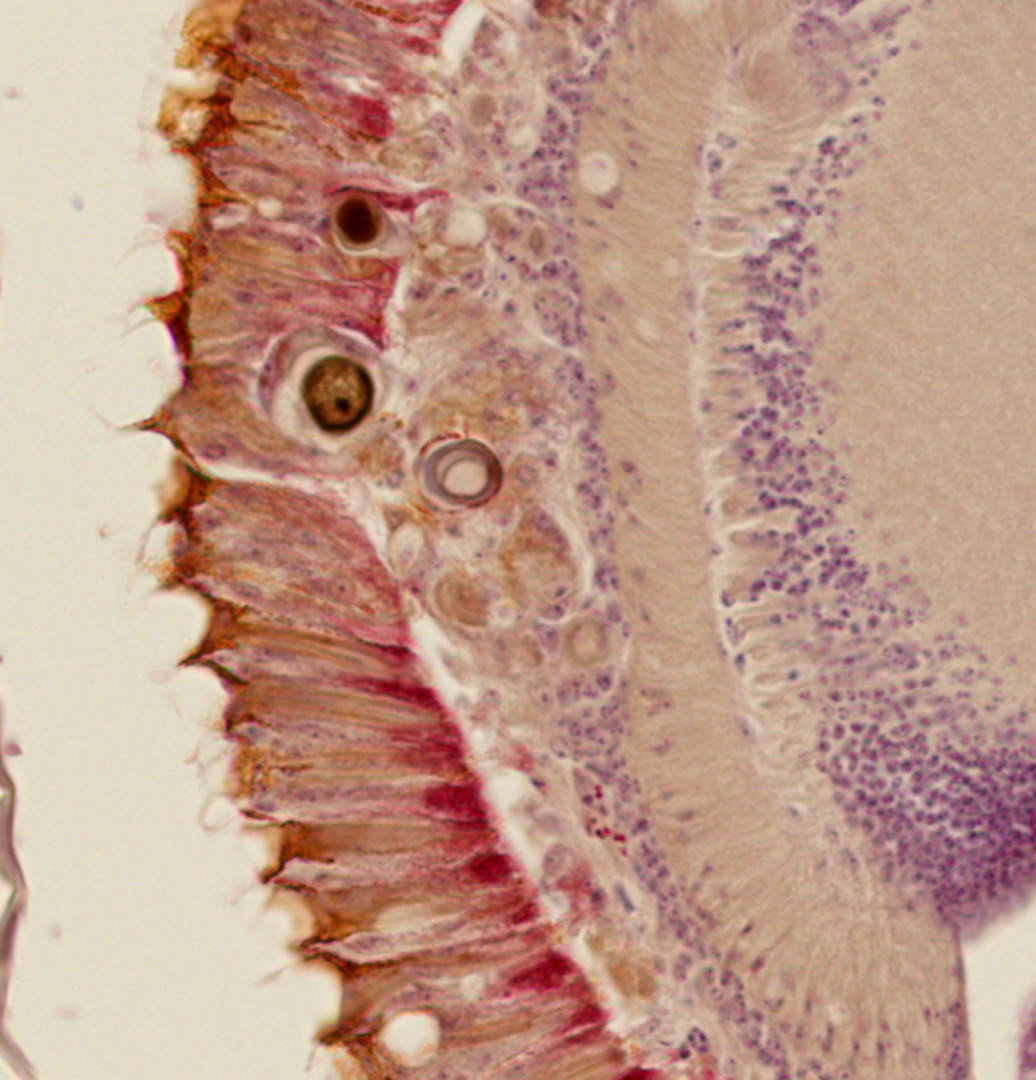
**Sectional image of a *Drosophila* retina expressing human Tau.** Tau expression in the retina causes photoreceptor degeneration indicated by vacuoles and abnormal inclusions which are mediated by glial phagocytosis. The retina was stained with haematoxylin and eosin.

**Why did you choose DMM for your paper?**

I always prefer to publish my results in Open Access journals. In our study, we discovered a new indicator for detecting hyperinflammation in the *Drosophila* retina expressing Tau. Therefore, I wanted to publish this study in a well-known journal specializing in disease modelling.

**Given your current role, what challenges do you face and what changes could improve the professional lives of other scientists in this role?**

As a postdoctoral researcher, managing time and focusing on the research theme has been challenging due to my diverse interests. While these multiple projects have allowed me to broaden my scientific scope beyond my initial interests, I now recognize the importance of concentrating on the primary theme. This focus will produce research outcomes that serve as a calling card, showcasing my expertise and paving the way for my future career as an independent researcher.

**What's next for you?**

Publish the papers I've been working on in my current lab and develop my own scientific narrative to become an independent researcher.

**Tell us something interesting about yourself that wouldn't be on your CV**

I've been into film photography since my college days. Recently, the prices for film camera gear have been going up, making it harder to keep up with the hobby. Nowadays, I mostly use a digital camera to make photo books when I travel to hidden places. But, my experience with film photography has really helped with my approach to microscopic imaging. I also love playing engine-building strategic board games and always aim to win.
